# *Brucella* spp. at the Wildlife-Livestock Interface: An Evolutionary Trajectory through a Livestock-to-Wildlife “Host Jump”?

**DOI:** 10.3390/vetsci5030081

**Published:** 2018-09-18

**Authors:** Jacques Godfroid

**Affiliations:** Department of Arctic and Marine Biology, Faculty of Biosciences, Fisheries and Economics, University of Tromsø-The Arctic University of Norway, Hansine Hansens veg 18, 9019 Tromsø, Norway; Jacques.godfroid@uit.no

**Keywords:** *Brucella* infection, wildlife/livestock/human interface, *Brucella*/host partnerships, host jump

## Abstract

*Brucella* infections in wildlife have gained a lot of interest from the scientific community and different stakeholders. These interests are often different and sometimes conflicting. As a result, different management perspectives and aims have been implemented (One Health, public health, veterinary public health, maintenance of a brucellosis free status in livestock, sustainable wildlife harvesting by hunters, wildlife and environmental health). When addressing *Brucella* infection in wildlife, the most important features of *Brucella* infection should be considered and the following questions need to be answered: (1) Is *Brucella* infection a result of a spillover from livestock or is it a sustainable infection in one or more wildlife host species? (2) Did the epidemiological situation of *Brucella* infection in wildlife change over time and, if so, what are the main drivers of change and does it impact the wildlife population dynamics? (3) Does *Brucella* infection in wildlife represent a reservoir of *Brucella* strains for livestock? (4) Is *Brucella* infection in wildlife of zoonotic concern? These questions point to the fundamental biological question of how animal (domestic and wildlife)/*Brucella* spp. partnerships are established. Will we be able to decipher an evolutionary trajectory through a livestock-to-wildlife “host jump”? Whole genome sequencing and new “omics” techniques will help in deciphering the molecular basis of *Brucella* host preference and open new avenues in brucellosis management aimed at preventing opportunities for *Brucella* host jumps.

## 1. Background: Different Perspectives of Brucellosis

### 1.1. The One Health Perspective

The One Health (OH) concept gained momentum by putting the emphasis on emerging zoonoses, particularly viral emerging zoonoses, with pandemic potential such as SARS and influenza in wildlife, livestock and at the wildlife/livestock/human interface [1]. Brucellosis is a bacterial disease, usually enzootic, that, in certain epidemiological situations, may show an increasing prevalence in livestock and/or wildlife, potentially leading to an increased incidence in human cases at a local or regional level. We understand intuitively that measures needed to mitigate the burden of zoonoses with pandemic potential, on one hand, and enzootic diseases like brucellosis, on the other hand, must be different [2]. Likewise, the degree of integration of different disciplines and sectors (public health, veterinary (public) health, environment health) will vary too. Indeed, in the case of an emerging zoonosis with pandemic potential, early detection in livestock and wildlife reservoirs is crucial to avoid transmission to humans leading to a pandemic, whereas from the public health perspective, early detection in the animal reservoir is of little relevance for brucellosis, which, if transmitted to humans, does not spread between humans (with the exception of occasional in utero transmission) and is thus of no pandemic concern.

### 1.2. The Disease Control/Eradication Perspective

In some developed countries, brucellosis in livestock (bovines, small ruminants, and pigs) has been eradicated and has thus become exotic or alien. From an economic point of view, it is of the utmost importance to reach and to keep a “brucellosis-free” status in livestock to be able to trade living animals freely without having to test animals for brucellosis before movement, which is extremely costly [3]. In this perspective, the presence of a reservoir of *Brucella* spp. in wildlife is, under certain conditions, considered a threat. Indeed, the transmission of *Brucella* spp. to livestock (“spillback” infection) may jeopardize the brucellosis-free status in livestock with disastrous economic consequences [4]. In this context, the management of brucellosis is done with the primary goal of preventing transmission from wildlife to livestock, and this is not done in an OH perspective. This is highlighted by *Brucella abortus* infection in cattle, bison (*Bison bison*), and elk (*Cervus canadensis*) in the Greater Yellowstone Area (GYA) in the USA [4]. Since all states in the USA are now designated “brucellosis-free”, the Designated Surveillance Area of Wyoming, Idaho, and Montana is essentially in a perpetual “Class A” status requiring that all sexually intact cattle leaving the area must be tested, vaccinated, and managed under other specific procedures and requirements [4]. Conversely, in Europe, there is a huge reservoir of *Brucella suis* biovar 2 in wild boar (*Sus scrofa*) that is not considered a threat for the pig industry and only of concern for outdoor reared pigs, a marginal production system [5]. As a result, there is no specific brucellosis management of brucellosis in wild boar, even more so given that *B. suis* biovar 2 is of limited zoonotic concern [6]. Recently, a focus of *Brucella melitensis* infection has been identified in a population of Alpine ibex (*Capra ibex*) in the Bargy area (French Alps), in a region where brucellosis was eradicated in livestock more than two decades ago. Interestingly, brucellosis in Alpine ibex has been identified after a human case associated with the consumption of cheese made from raw cow milk had been diagnosed. The tracing back to the *B. melitensis*-infected cattle herd and the molecular typing of *Brucella* isolates suggested that the bovine *B. melitensis* infection could have originated from the Alpine ibex population [7]. However, from a veterinary and public health perspective, the risk of spillback to livestock and from there to humans is considered very low [8].

### 1.3. The Public Health Perspective

In low- and middle-income countries (LMICs), brucellosis in livestock is to a large extent still enzootic and it is in these countries that human patients suffer the most important burden of zoonotic brucellosis. Out of the 500,000 new human brucellosis cases occurring every year, the vast majority is occurring in LMICs and is connected to contact with infected livestock and consumption of infected animal products [9]. In sub-Saharan Africa, no proof of direct transmission of *Brucella* spp. form wildlife to humans has been reported in the international scientific literature, although transmission from preparing and consuming buffalo (*Syncerus caffer*) bushmeat has been suggested [10]. A recent systematic review on wildlife brucellosis in Africa highlights that buffalo is by far the wildlife species in which brucellosis has been most frequently reported, mainly through serological inquiries, and only rarely by isolation of *Brucella* spp. [11]. There is therefore a need to isolate, identify, and characterize *Brucella* strains from buffalo and human patients to be able to demonstrate direct transmission from wildlife to humans. This would alert public health officials to human *Brucella* infections arising from the consumption of bushmeat.

Special attention needs to be given to *Brucella* infections in the Arctic. Indigenous people have lived in the Arctic for thousands of years. Nowadays, the region represents one of the least populated areas in the world, with sparse nomadic communities and very few large cities and towns. Residents of the Arctic include several indigenous groups as well as more recent arrivals from more southern latitudes. In total, only about 4 million people live in the Arctic, and in most countries indigenous people make up a minority (about 10%) of the Arctic population. Once completely nomadic, indigenous people are now mostly sedentary or seminomadic, practicing traditional methods of hunting, fishing, reindeer herding, gathering wild plants for food, and indigenous arts and crafts. The intergovernmental Arctic Council (https://arctic-council.org/index.php/en/) promotes sustainable development in the Arctic region, including economic and social development, improved health conditions, and cultural well-being for Arctic people. Very little livestock farming occurs in the Arctic, and thus the origin of human brucellosis is to be found in Arctic wildlife. Up to this day, only *B. suis* biovar 4 infection originating in semi-domesticated reindeer (*Rangifer tarandus*) has been diagnosed in human patients [12], with no known human case of *Brucella* infection originating from other terrestrial wildlife species or marine mammals [13].

## 2. Infection Biology and Serological Diagnosis of Brucellosis

As for any type of zoonosis at the wildlife/livestock/human interface, there is a need to decipher and understand the infection biology of *Brucella* spp., the ecology of *Brucella* infection in multi-host complex systems, and the drivers of change in the epidemiology of *Brucella* infection to be able to design and implement valid mitigation strategies. It is important to acknowledge that such strategies have different aims and will thus be implemented in different perspectives, including or not OH. These different aims (OH, public health, veterinary public health, maintenance of a brucellosis-free status in livestock, sustainable wildlife harvesting by hunters, wildlife and environmental health) must be clearly highlighted beforehand to allow stakeholders to take ownership, avoiding discipline-specific “silos” that only rarely interact with one another and are likely to (at best) only partially address the concerns of some interested groups [14].

Before addressing transmission of *Brucella* spp. at the wildlife/livestock/human interface, it is worth being reminded of some of the most important features of *Brucella* infection biology and diagnostics [15,16].

*Brucella* exposure may or may not be followed by *Brucella* infection. *Brucella* infection may or may not induce a protracted immune response resulting in local or general pathology and in the production of antibodies, detectable by serology. Pathology may or may not result in clinical signs. When *Brucella* infection results in clinical signs, it is named brucellosis. Brucellosis is characterized by acute infections (e.g., abortion) and chronic infections (e.g., hygromas).

Animals infected with *Brucella* spp. are only infectious to congeners, other animal host species and humans at limited periods of time. Typically, aborting females are a source of bacteria through aborted fetuses, vaginal discharges (lochia), and milk. Males may intermittently excrete *Brucella* spp. in semen (venereal transmission) and have not been shown to be a significant source of infection except for *Brucella ovis* and to a lesser extent *Brucella suis*. In other words, *Brucella*-infected animals are not always infectious and thus transmission cannot occur outside a time window when animals excrete *Brucella* spp.

Besides being 100% sensitive and 100% specific, an ideal serological test should make it possible to differentiate infectious animals from infected or exposed ones. Unfortunately, such a test does not exist, and the only diagnosis of certainty is bacterial isolation or DNA detection of *Brucella* spp. Brucellosis serology has other drawbacks, among which is the impossibility to ascribe which *Brucella* species induced antibodies in the host and the impossibility (per definition) to detect “latent” infection, defined as a seronegative *Brucella*-infected animal. Latent infection has been well studied in bovines, where up to 10% of calves born to *B. abortus*-infected heifers remain seronegative [17]. Most latently infected female calves will abort at their first pregnancy. Latent infection has not been studied in wildlife brucellosis and thus brucellosis seroprevalence studies in wildlife may be biased and possibly misleading.

## 3. Transmission of *Brucella* spp. at the Wildlife/Livestock/Human Interface

The aim of this manuscript is to highlight some of the questions that need to be addressed when studying *Brucella* infections in wildlife and at the wildlife/livestock/human interface.

### 3.1. Is *Brucella* Infection a Result of a Spillover from Livestock or Is It a Sustainable Infection in One or More Wildlife Host Species?

One of the hallmarks of *Brucella* infection is host specificity or preference, given that specificity is not absolute. For livestock species, it is well recognized that *B. melitensis* infects primary members of the family Bovidae, subfamily Caprinae, like sheep (*Ovis aries*) and goats (*Capra aegagrus hircus*). These host species are “reservoir” host species, i.e., the host in which the infectious agent multiplies and/or develops, and on which the agent depends for survival in nature. However, members of the family Bovidae, subfamily Bovinae, like cattle (*Bos taurus*), and members of the family Camelidae, like dromedary camels (*Camelus dromedaries*), can also be infected and show typical brucellosis clinical signs like abortion. Importantly, *B. melitensis* infection is always reported in cattle and camels having had contact with *B. melitensis*-infected sheep and goats. This suggests that under traditional husbandry systems, cattle and camels may not be *B. melitensis* sustainable hosts, i.e., able to maintain the infection within their species without any external source of bacteria from a reservoir host species.

There is ample information in the literature highlighting that different wildlife species have been exposed to *Brucella* spp. However, very few studies address the sustainability of *Brucella* infection in wildlife. Those *Brucella* infections that are recognized as sustainable in wildlife are the following ones: *Brucella abortus* in buffalo and bison; *B. melitensis* in Alpine ibex; *Brucella suis* biovar 2 in wild boar and European hare (*Lepus europeaus*); *Brucella suis* biovar 4 in reindeer (*Rangifer tarandus*); *Brucella ceti* in cetaceans. Recently, new species of *Brucella* have been described in different host species, i.e., *Brucella microti* in voles and red fox (*Vulpes vulpes*); *Brucella vulpis* in red fox; *Brucella inopinata* in frogs and humans; and *Brucella papionis* in baboons (*Papio* spp.) [18]. However, it is still not known if these hosts are the (sole) preferential hosts. *Brucella suis* biovar 1 infection in feral pigs in the USA and Australia will not be discussed in this manuscript, since feral pigs are pigs living in the wild but which have descended from escaped or released domesticated individuals, some of which were infected with *B. suis* biovar 1.

One of the most intriguing features of brucellosis in wildlife concerns *B. abortus* infection in members of the family Cervidae, subfamily Cervinae, i.e., red deer (*Cervus elaphus*) in Europe and elk (*Cervus canadensis*) in North America, now classified as two different species.

There are only anecdotal reports on *B. abortus* infection in red deer in Europe, even at the time *B. abortus* was enzootic in cattle populations throughout the continent, suggesting that red deer are not able to sustain *B. abortus* infection, under management practices implemented in Europe [19]. The situation is dramatically different in some elk populations in North America, as highlighted in the GYA. Indeed, whole genome sequencing of *B. abortus* isolates collected from 1985 to 2013 in cattle, elk, and bison across the GYA suggest that *Brucella* infection was introduced into GYA bison and elk on at least five separate occasions, presumably from cattle. One of these five clades is mainly associated with bison within Yellowstone [20]. However, these estimates only provide the number of transition events for the currently available isolates rather than estimating actual transmission rates per unit time in different locations and thus the amount of cross-species transmissions that occurs between elk and bison is currently unknown [20]. Nevertheless, this suggests that there may be significant differences in host preference among different *B. abortus* clades. Likewise, it is important to recognize that there has been limited progress in understanding *Brucella* host preference and genetic resistance to brucellosis [21]. So far, the identified variations only partially explain the differences in virulence among *Brucella* species and their specificity for certain host species [22].

The *B. abortus* isolates from many of the unfed elk in Montana and Wyoming, however, originated from the Wyoming feedgrounds instead of Yellowstone bison. Two different clades were able to move from Wyoming feedgrounds to western Montana, potentially in the 1990s to early 2000s, followed by subsequent local transmission rather than repeated invasions from the feedgrounds, suggesting that elk can maintain the infection locally after those introductions [20].

### 3.2. Did the Epidemiological Situation of Brucella Infection in Wildlife Change Over Time and, If So, What Are the Main Drivers of Change and Does It Impact the Population Dynamics?

In October 2017, a technical report entitled “Revisiting Brucellosis in the Greater Yellow Stone Area” was published by the US National Academies Press [4]. In its preface, the following is mentioned: “This report examines the changing dynamic of brucellosis in the GYA, providing a comprehensive update of what is new since the 1998 National Research Council report “*Brucellosis in the Greater Yellowstone Area”* and exploring various options for addressing the challenge of brucellosis disease management. Much has changed in the 19 years since the previous report. There is now clear evidence that transmission of *B. abortus* to domestic livestock in the GYA has come from infected elk, not bison, posing greater challenges for control of transmission to domestic species”.

The GYA is estimated to support roughly 450,000 cattle that have the potential to come into contact with approximately 125,000 elk and 3000 to 6000 bison residing in the GYA. Since 2000, 27 outbreaks of *B. abortus* infection in cattle and domestic bison have been detected in the GYA, and all these outbreaks have originated from spillback *B. abortus* infection from elk, as demonstrated by molecular tools [20]. Why did these changes occur at this point in time and what are the drivers of such dramatic changes? Ecological changes within the GYA since 1998 have shifted the dynamics of wildlife populations. For instance, “the reintroduction of wolves and increases in grizzly bear numbers have impacted the density and distribution of elk, and the rising number of private landowners has changed land use around national parks, with private lands increasingly serving as refugia for elk from hunting” [4]. The reader is referred to this publication for information related to management options, which will not be discussed in this manuscript.

Importantly, it is not known if these ecological changes were sufficient to allow some *B. abortus* clades to overcome host species barriers, and such changes do not explain how unfed elk populations can maintain brucellosis at seroprevalence levels like those of elk on the supplemental feedgrounds in Wyoming [20]. While the possibility of a host jump of a *B. abortus* clade to GYA elk is an appealing hypothesis, it still needs to be tested.

Although *B. abortus* induces abortion events and has the potential to have significant impacts on individual animals (such as testicular abscesses, retained placentas, arthritis, death of neonates), it is not generally considered to be a direct threat to the sustainability of either elk or bison populations [4]. The same conclusions have been obtained in *B. abortus*-infected buffalo in Kruger National Park, South Africa [23].

### 3.3. Does Brucella Infection in Wildlife Represent a Reservoir of Brucella Strains for Livestock?

Different answers are given to this question, as highlighted by the transmission of *B. abortus* in the GYA between elk, bison, and cattle. Interestingly, since 2000, only spillbacks from elk have been reported. No spillback from bison has been documented, notwithstanding that the prevalence in bison is comparable to the prevalence before 2000, highlighting that the spatial and temporal separation management between cattle and bison (still considered a threat) is a sound management practice in the GYA.

In Europe, only a few cases of *B. suis* biovar 2 transmission from wild boar to outdoor-reared pigs and bovines have been documented. In the latter case, there is clear evidence that bovines cannot sustain *B. suis* biovar 2 infection [24].

In Kenya, a recent serological inquiry performed on cattle from three villages at varying distances from the Maasai Mara National Reserve, home of large populations of wild ruminants such as buffaloes and wildebeest (*Connochaetes taurinus*), suggested that closer contact with wildlife may increase the transmission of *Brucella* spp. between livestock and wildlife [25]. Comparable results were obtained at selected livestock/wildlife interface areas of the Gonarezhou National Park, Zimbabwe [26]. A recent study performed in South Africa in a rural community established at the border of Kruger National Park (home of more than 35,000 buffaloes with an estimated brucellosis seroprevalence of 10%) suggested that the close proximity of brucellosis-infected buffalo was not a threat to domestic animals in a controlled setting with vaccination, fencing, and movement control [27].

These examples highlight that every single situation is a study case in its own right, and that no general unambiguous answer should be given.

### 3.4. Is Brucella Infection in Wildlife of Zoonotic Concern?

It is worth noting that, to date, there is no documented report on the direct transmission of *B. abortus* from elk to human in the GYA in the scientific literature. Although the Yellowstone Park service raises awareness about the risk for people in the GYA (https://www.nps.gov/yell/learn/nature/brucellosis.htm), the US Center for Disease Control and Prevention only mentions hunting feral pigs (infected with *B. suis* biovar 1) as a zoonotic risk (https://www.cdc.gov/brucellosis/exposure/hunters.html).

The best way to answer this question unambiguously is to analyze the situation in the Arctic, where livestock farming is almost nonexistent. In the Arctic, *B. suis* biovar 4 infection is found in reindeer/caribou, and *Brucella ceti* and *Brucella pinnipedialis* infections are found primarily in different cetacean and seal species, respectively. Human *B. suis* biovar 4 infections originating in semi-domesticated reindeer have been reported in indigenous people in Canada, Alaska, and Russia. In North America, the average number of cases is one per year [12]. The situation is globally comparable in Russia, with an exception of Yakutia, in the Far East, where the percent of infection remains high (4.8–5.6%) among reindeer breeders [28].

Although *Brucella* infection is widely spread in cetaceans and pinnipeds in the Arctic, does this mean that there is a zoonotic risk? *Brucella pinnipedialis* Strain Type 27 (ST27) is the only marine mammal *Brucella* strain that has been documented in natural infection cases in humans [29,30]. To date, less than five cases have been reported worldwide and all of them occurred in the Southern hemisphere [31]. Some of these cases were not related to contact with marine mammals or consumption of their products, suggesting that the infection may have been acquired through the consumption of seafood [29]. Interestingly, it has been documented that true seals are likely *Brucella* spillover hosts [32,33], acquiring the infection from fish [34] or lungworms [35], while the infection biology in eared seals is not known [13]. In the Arctic, ST27 has, to date, not been isolated from any marine mammal species.

Although upholding a traditional life style may represent a risk for *Brucella* infection in indigenous people in the Arctic, the risk is very low for *B. suis* biovar 4 (with the exception of Yakutia, Russia) and negligible for *B. pinnipedialis* ST27. Under such circumstances, the issue becomes how to communicate brucellosis risk while still promoting the health benefits of a traditional life style. In this perspective, the Alaska Native Tribal Health Consortium has published information about brucellosis, addressing frequently asked questions by indigenous people (https://anthc.org/wp-content/uploads/2016/01/CCH-Bulletin-No-6-Brucellosis.pdf).

## 4. Conclusions

Regardless of health and management perspectives, the aforementioned questions and answers point to the fundamental biological question of how animal (domestic and wildlife)/*Brucella* spp. partnerships are established. In 2015, it was reported that the evolutionary trajectory of the common rabbit clone of *Staphylococcus aureus* evolved through a likely human-to-rabbit “host jump” some 40 years ago and that only a single naturally occurring nucleotide mutation was required and sufficient to convert a human-specific *S. aureus* strain into one that could infect rabbits [36]. Such a single naturally occurring mutation associated with a bacterial host-switching event represents a paradigm shift in the understanding of the minimal adaptations required for a bacterium to overcome species barriers and establish itself in new host populations.

The capacity of microbial pathogens to alter their host tropism in distinct host species populations is a global public and veterinary health concern. Ecosystems are complex multi-host systems. A pathogen host jump in such systems will occur after selection of naturally occurring mutants by the multiplication of contacts, with only a few of them being infectious. The multiplication of contacts at the wildlife/livestock/human interface resulting in emerging diseases is mainly the result of anthropological-driven ecological changes [37]. In the case of the GYA, it is now accepted that feedgrounds have played a major role in the changes occurring in the epidemiology of brucellosis at the elk/bison/bovine interface [4]. Following such ecological changes, we hypothesize that nucleotide mutation events occurring naturally in *Brucella* strains may have been selected by the multiplication of exposure of elk to *B. abortus* strains originating in cattle, resulting in a host-switch converting a few bovine *B. abortus*-adapted strains to strains that could sustain *B. abortus* infection in elk. Such a hypothesis needs to be tested. Recently, it has been demonstrated that *Mycoplama gallisepticum* emergence in house finches (*Haemorhous mexicanus*) requires more than direct contact with domestic poultry. Indeed, mutations arising in its original poultry host are necessary for successful pathogen emergence in the novel finch host [38]. Whole genomic sequencing and analysis by new “omics” techniques of *B. abortus* isolates from different host species before and after the year 2000 may highlight whether *B. abortus* in the GYA evolved through a bovine-to-elk host jump. Such studies will help in deciphering the molecular basis of *Brucella* host preference and open new avenues in brucellosis management aimed at preventing opportunities for *Brucella* host jumps.
